# Computational Fluid Dynamics Analysis in Biomimetics Applications: A Review from Aerospace Engineering Perspective

**DOI:** 10.3390/biomimetics8030319

**Published:** 2023-07-20

**Authors:** Ernnie Illyani Basri, Adi Azriff Basri, Kamarul Arifin Ahmad

**Affiliations:** Department of Aerospace Engineering, Universiti Putra Malaysia, Serdang 43400, Selangor Darul Ehsan, Malaysia; adiazriff@upm.edu.my

**Keywords:** Computational Fluid Dynamics (CFD), biomimetics applications, mimicking nature, aerodynamics

## Abstract

In many modern engineering fields, computational fluid dynamics (CFD) has been adopted as a methodology to solve complex problems. CFD is becoming a key component in developing updated designs and optimization through computational simulations, resulting in lower operating costs and enhanced efficiency. Even though the biomimetics application is complex in adapting nature to inspire new capabilities for exciting future technologies, the recent CFD in biomimetics is more accessible and practicable due to the availability of high-performance hardware and software with advances in computer sciences. Many simulations and experimental results have been used to study the analyses in biomimetics applications, particularly those related to aerospace engineering. There are numerous examples of biomimetic successes that involve making simple copies, such as the use of fins for swimming or the mastery of flying, which became possible only after the principles of aerodynamics were better understood. Therefore, this review discusses the essential methodology of CFD as a reliable tool for researchers in understanding the technology inspired by nature and an outlook for potential development through simulations. CFD plays a major role as decision support prior to undertaking a real commitment to execute any design inspired by nature and providing the direction to develop new capabilities of technologies.

## 1. Introduction

Through the evolution of technology, adapting the idea from biology, creatures in nature were viewed as engineering designs with general features. It has directed the pool of inventions towards an increasingly enhanced potential of their capabilities towards engineering capabilities, tools, and mechanisms [1,2]. Hence, our technology significantly emerged by adapting the features and characteristics of nature’s capabilities, which are far superior to human capabilities in many areas. This term, known as ‘biomimetics’,” implied the study of imitating or adapting nature’s mechanisms, methods, and processes. It was coined by Otto H. Schmitt [3]. Biomimetic technology emerged from innovative ideas from the biological sciences into engineering applications that inspired humans for thousands of years, and the results have benefited the quality of life, improved surviving generations, and secured a sustainable future. Through the principles of mechanics, chemistry, physics, materials science, control, mobility, sensors, and other engineering and science fields, this transfer function from nature’s capabilities to artificial devices has drawn the attention of a novel research agenda across various disciplines [2]. The emerging field of biomimetics has also concerned scaling from nano and micro to macro and mega through the integration of biology, natural history, and material sciences [4].

From the perspective of engineering designs, creatures in nature have an enormous pool of creations and inventions that offer boundless potential and motivate new capabilities for thrilling future technologies. There are numerous examples of biomimetic successes that involve exact duplicates, such as the use of fins for swimming and efficient propulsors, greater mimicking of the mastery of flying for understanding the principles of aerodynamics, imitating the biological honeycomb for structural configuration of low weight and high strength, and many more. Adapting nature’s capabilities into engineering capabilities, including tools and mechanisms, required a remarkable effort to critically bridge both fields of biology and engineering. Interestingly, significant progress has been made with biologically inspired capabilities, which are becoming more sensible and possible for man-made technologies, including artificial intelligence (AI), biologically inspired mechanisms, biologically inspired structures and tools, biological materials, biosensors, etc. Captivatingly, the greatest challenge in biomimetics is mimicking nature’s capabilities to create devices of miniature size, considering various aspects of biology that are still beyond human understanding.

Inspired by biological mechanisms, research shows that significant advances in computer-based simulation are growing rapidly in accordance with their importance and rapid acceptance for applications in biomimetics. Computational Fluid Dynamics (CFD) is one of the widely adopted methodologies of computer-based simulation, which is defined as a branch of fluid dynamics that uses numerical solutions of the governing equations for simulating real fluid flows [5]. CFD is becoming a key component in developing updated designs and optimizing them through computational simulations. However, recent CFD is still emerging in biomimetics applications due to the complexity of the anatomy and fluid behavior of creatures in nature. Nevertheless, it is becoming more accessible and practicable by virtue of the advent of digital computers with high-performance hardware and software [6]. Since the importance of knowledge of body fluids and system components in fluid flow studies has been growing over the last several years, the advancement of biomimetics practices and technology has been stimulated. The research of biomimetics with the aid of CFD software is still emerging and incorporates mechanisms of biologically inspired capabilities through simulation.

To date, CFD is increasingly applied in a wide range of critical engineering systems, incorporating an expert area of mathematics and a branch of fluid mechanics. CFD modeling has already received tremendous attention from biomimetics research, along with the development of biologically inspired technologies. Furthermore, detailed characterization of complex biological features and the measurement of computation metrics can be determined by incorporating both design features and CFD simulation. Therefore, this paper explores the CFD study using the state-of-the-art in the aeronautical area, highlighting the biomimetics applications.

## 2. Adaptive Bio-Inspired Applications in Aircraft Technology

In the field of aircraft technology, the basic inspiration and motivation for flying have come from the capabilities of birds, insects, and aquatic animals that are able to generate efficient lift and thrust with the same wing planform. This bio-inspirations attempt attempts to produce engineered systems that possess characteristics in aeronautical applications, in which this has inspired humans towards replicating or mimicking the features and capabilities of the biological evolution in human-engineered systems. The notion of characterizing animal features is far from new. Leonardo da Vinci, the first to develop early blueprints for a “flying machine’ inspired by a bird, adopted a flapping mechanism to produce lift and thrust [2]. Then, the Wright Brothers succeeded in creating and flying the first airplane off the ground by adapting the ability of a pigeon’s wing to create lift. Throughout biological evolution, the increasing demand for integrating the structure and functions to replicate the features of animal species has driven designers towards simpler and more efficient designs; hence, significant progress has been made. Namely, one can take biologically identified anatomical structures and their functions in engineering applications, as in Table 1.

**Figure 1:**
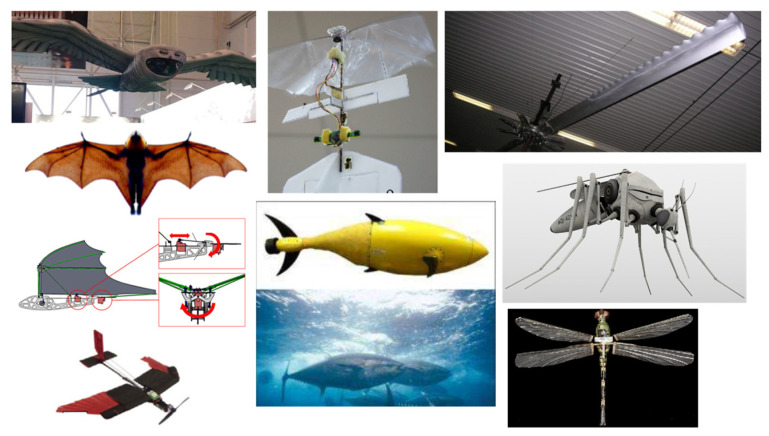
Examples of bio-inspired animals in engineering applications.

Referring to Table 1, the basic motivation for flying has always come from millions of species of birds and insects. They efficiently generate lift and thrust using the same wing planform. The specialized feathers of the owl have the ability to fly silently with their unique wing features of trailing edge fringe and velvety down that help absorb aerodynamic sound [7], whereas the morphology of insects is way more complex, which aerodynamically produces pressure gradients for lift and thrust by flapping from multiple wings and legs. In fact, dragonflies inspired the idea to build four-winged MAVs [9]. Despite that, the limbs of bats also influenced flight performance, such as the geometry of wings and bones, compliant skin and bones, distribution of sensory hairs across wings, and the physiology of the musculature that drives the wings [9,12]. In the late 1990s, Frank E. Fish discovered the tubercle effect of the flipper of the humpback whale, which acts like a wing and contributes to superior aerodynamic maneuverability that allows greater lift and less drag than a smooth surface fin [15]. The humpback whale flipper received tremendous attention for the influence of rounded tubercles located on the leading edge of flippers in order to design effective wings involving aerodynamic performance [14,15,16,17,18]. Figure 1 shows examples of bio-inspired animals in engineering applications.

Increasingly, research on simulations with regard to aerodynamic or hydrodynamic studies of biologically inspired capabilities is particularly focused on the anatomical structures of animal features. CFD modeling has received remarkable interest among researchers, along with the development of technological devices through these inspirations, especially in the aerospace field.

## 3. Research on Biomimetics Related to CFD Applications

CFD plays an important role by offering chances for simulation prior to undertaking a real commitment to develop biologically inspired innovations using the correct methods and to execute any design alteration. The research of biomimetics CFD applications related to the aerospace field has drawn attention in the past few years due to the importance of computational simulations in order to evaluate aerodynamic behavior such as aerodynamic efficiency, lift enhancement, drag reduction, and flow separation control. The CFD approach is used to understand the low regime by taking into account the Reynolds number (*Re*) in order to have a deep understanding of the nature of the application. The biomimetics CFD applications for animals’ anatomical structures are discussed in the subsequent sub-sections.

### 3.1. Biological Organisms of Plant Species

**Figure 2:**
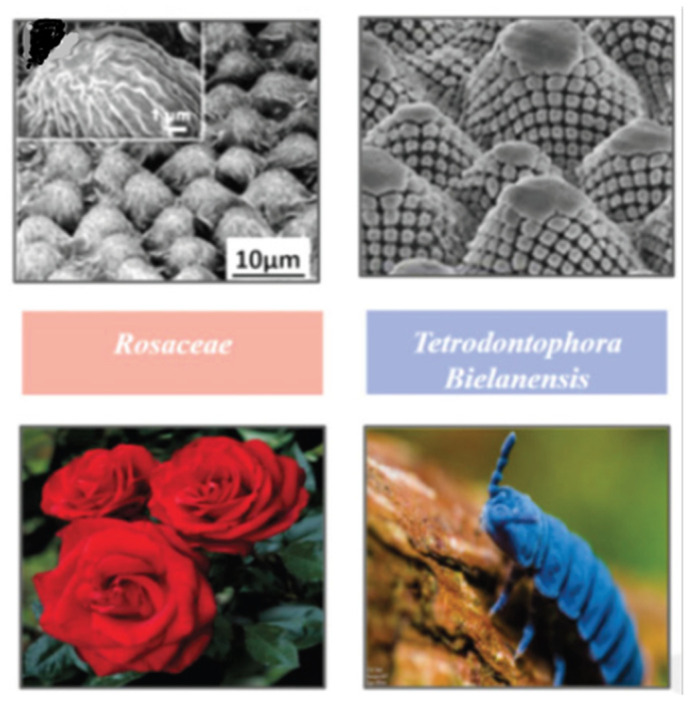
Morphological structures of biological organisms (Source: [23]).

In the basic structure of biologically inspired capabilities, one of the motivations for the research is to study the behavior of certain plant species with the characteristic of being super-hydrophobic, which is associated with skin friction drag reduction quality. Rose et al. [22] highlighted that most research in regards to bio-inspiration applications of plant species mostly focused on experimental studies to understand the novel insights on aerodynamics, as in Figure 2. In a recent study, Karthik [23] studied the fluid flow over a surface of the morphological structures of biological organisms, namely Tetrodontophora bielanesis, and Rosaceae, by using one of the CFD software packages known as OpenFOAM. In the study, the authors performed fluid flows over a surface where there was a lot of vortex shedding, which reduced the drag force compared to a planar surface. From the results, the morphological structure of Tetrodontophora bielanesis was presented as being the most efficient in terms of drag reduction, with 83% drag force reduced at a freestream velocity of 1 m/s compared to the flat surface. Due to the parabolic structures of Tetrodontophora bielanesis and Rosaceae, better drag reduction was observed. As the loss of energy can be minimized by drag, this hydrophobic study has a commercial interest in designing aerospace vehicles. Besides drag reduction, higher self-cleaning efficiency, and anti-fouling can be exhibited by the characteristics of plant surfaces [24]. There were fewer studies available in performing CFD simulations to understand the drag reduction induced by the plant surfaces, which benefited the further development of aerodynamic parts of aircraft.

### 3.2. Insects

In general, flapping flight mechanisms are commonly adapted by insects subjected to their functions. However, insects like butterflies and dragonflies spend most of their time gliding, and this behavior of gliding can be compared to the flow over an airfoil used in the conventional lift generation of aircraft through CFD simulation. By replicating natural body features and approaches with biomimetic applications, this could potentially contribute to the modern drone or robot industry.

**Figure 3:**
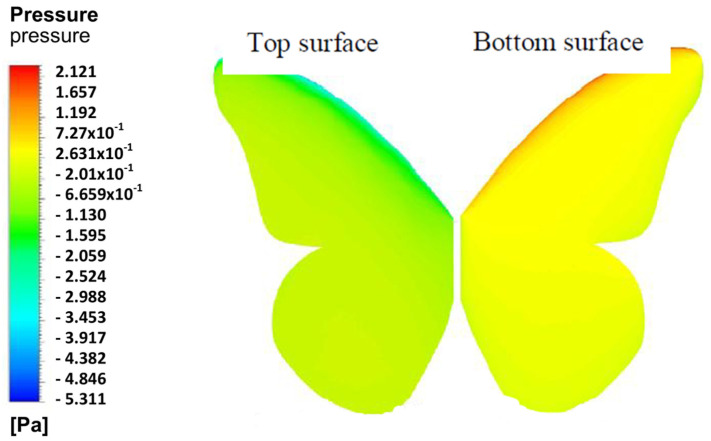
Example of pressure result on Monarch butterfly using CFD (Source: [30]).

Kumar and Pendyala [25] studied the aerodynamic performance of a Monarch butterfly’s “Danaus plexippus” wing using ANSYS CFX software, as shown in Figure 3. The authors performed the CFD simulation to understand the behavior of a gliding butterfly subjected to two orientations (open and closed wing) at three different velocities of 3 m/s, 4 m/s, and 6 m/s and the variation of the angle of attack (AoA). The greatest improvement was found at AoA 6.8 degrees with a maximum *Cl*/*Cd* ratio. Considering the low *Re* numbers, there is a possibility that Re increased at higher speeds with less pressure drag and separation. Thus, these factors are supposed to be considered during the design process of replicating the butterfly forewing-inspired geometry for the applications of the Micro-Air Vehicle (MAV). Hence, this MAV is able to be extended further to improve endurance range and maximum speed and then positioned forward to increase lift at high AoA so that better aerodynamic efficiency can be achieved. Another study on butterfly wings by Zhang et al. [26], investigated the morphological performance of the butterfly species Chilasa clytia. The authors built the 3D butterfly wing model of the forewing and hindwing prior to performing CFD simulation using XFlow software, which was combined with ADAMS software for kinematics calculation. The high coupling of the body and wing motion is the main element for lift generation and altitude adjustment during forward flight. From the simulation, the average lift and drag generated by a flapping wing increase synchronously with flight velocity, so the high values of instantaneous aerodynamic forces increase accordingly. Besides that, Ghosh [27] performed CFD analysis using ANSYS Fluent software to the flight of a butterfly for MAV applications. The author conducted a 2D flapping wing using dynamic mesh methods from the PIV data of the motion of the wings from a previous study [28]. From the result, it can be observed that a significant amount of lift was generated due to the lift produced by the flapping wing coupled with the body movement, with a Cl coefficient of 0.0021. The aerodynamic behavior of lift improvement and drag reduction was achieved throughout the study. Sanuki and Fujikawa [29] study the motion analysis of a butterfly-style flapping robot by comparing CFD and experiments. The authors performed fluid-structure interaction studies, including multibody dynamic simulation and thermo-fluid analysis, using hardware from MotionSolve^®^ and AcuSolve^®^ and software from HyperWorks^®^ for CFD. From the study, it can be observed that there is no significant difference in maximum lift and only a minimum value for pitch angle. However, the maximum value for body pitch moment showed a twofold increase for a higher stroke value of 0.270 mNm. Negative pressure generated on the upper surface of the wing during downstroke and negative pressure found on the lower part of the wing during upstroke. The authors concluded that the flying insects produce high lift effectively through separation vortices subjected to the shape of the wings and body.

On the other hand, another insect of interest that inspired the development of MAV is the mosquito, yet less study is available to understand aerodynamic behavior using CFD simulation. Bomphrey et al. [31] performed CFD analysis and compared it numerically with the blade element model with the quasi-steady assumption of a hovering mosquito’s wing to determine the force coefficients of *Cl* and *Cd* at *Re* numbers 50 to 300. Singh et al. [11] presented the review studies on the bioinspired mosquito to mimic the role of robotic systems in miniature UAVs. The authors highlighted the unique unsteady aerodynamic characteristic of mosquito flight, which should include rotational drag, wake capture reinforcement of the trailing edge vortex, and an added mass effect for flight in a low-density environment. In order to understand the dynamic of small insects like mosquitoes mimicking robots, CFD is suggested as a great tool to generate the aerodynamic force and moment in an oscillatory condition because of unsteady flow.

**Figure 4:**
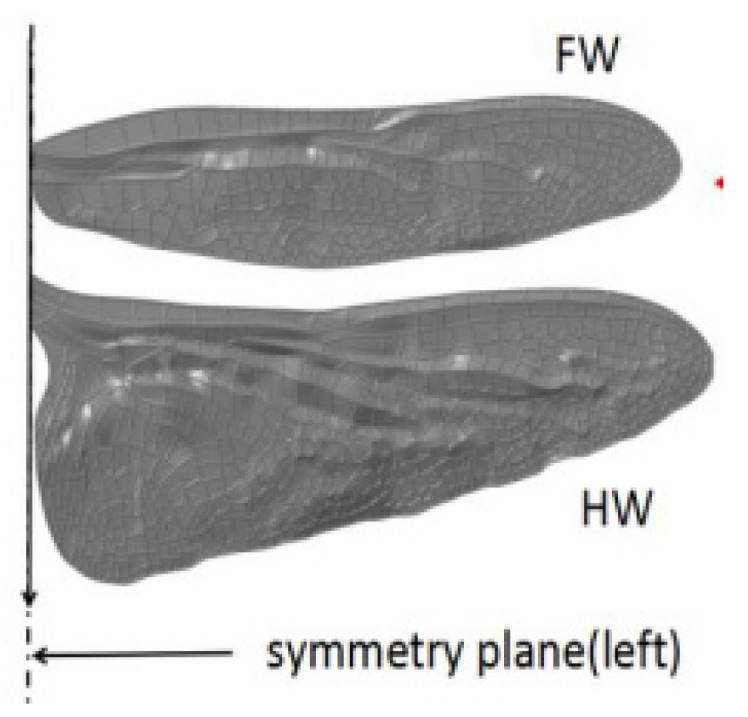
Forewing and hindwing of a dragonfly (Source: [32]).

Besides that, dragonflies can be another type of insect of interest, as shown in Figure 4. Luo et al. [32] studied the aerodynamic performance of dragonfly forewing-hindwing interactions subjected to gliding conditions. By using the CFD technique, the authors showed that the effects of dragonfly forewing-hindwing interaction at gliding motion correspond to the changes in AoA from 0° to 25°. According to the study, the effect of forewing contributed to a significant aerodynamic effect at small AoA. A. Prasad et al. [33] carried out a CFD analysis on a geometry model of a dragonfly flapping wing model frame for the application of nanoair vehicles (NAV). With the aid of ANSYS Fluent software, the authors aimed to analyze the aerodynamic forces and movement acted on a flapping wing at a low *Re* number. It can be noticed that lift increased linearly with the angle until the stall, with *Cl* of 0.02035 and *Cd* of 0.3109. Khan and Phady [34] focused on the variation of corrugation angle and peak height subjected to AoA of a 2D corrugated airfoil, which was inspired by the yellow dragonfly basal wing section. By using ANSYS Workbench, the authors performed a CFD simulation comparing the design of the wing based on corrugation angle, pitch length, and peak height. The result of aerodynamic performance showed that the bio-inspired wing provided better and more consistent results. The formation of separation bubbles is found because of an adverse pressure gradient, which affected the recirculation zone inside the cavities, and the flow reattached upstream of the subsequent corrugation. Inspired by the flapping wing of a dragonfly, Shern et al. [35] investigated the aerodynamic performance of a MAV based on perturbed flow conditions by using the ANSYS Fluent CFD technique. The simulation was conducted after validating the CFD study with previous works on CFD simulation and experiment and obtaining good agreement in terms of lift coefficient. The authors further the study of fluid dynamic behavior by examining the existence of vortex or flow vorticity subjected to AoA and wind disturbance. The results proved that MAV is best operated at an AoA of 8° due to maximum *L/D* with and without the presence of wind disturbances. Luo et al. [32] conducted a CFD simulation to study the aerodynamic performance of a 3D model dragonfly with corrugation of forewing-hindwing interaction in gliding flight subjected to AoA from 0° to 25°. As a result, the aerodynamic performance of the forewing is slightly better than that of the hindwing at a small AoA. The forewing-hindwing interaction increases the lift coefficient of the forewing significantly and has a growth rate of 17.1%, 11.4%, 17.7%, 25%, 31.1%, and 30.7%. Zhou et al. [36] performed two simulations of CFD and computational structural dynamics (CSD) to verify that the structure configurations (flexible and rigid) of a MAV wing design influence the aerodynamics of the airfoil. Inspired by natural flyers, the author conducted the simulation on the 2D wing design by simulating the aerodynamic forces acted on the airfoils and further computed the transient analysis of CSD to determine the deformation of the airfoil by using ANSYS. The authors observed that the flexible airfoil results in lower drag with a maximum lift of 2.8 times better than that of a rigid one and is fully deformable, which improves its aerodynamic performance.

Another insect-like flapping wing that received attention from Nakata et al. [37] was the hawkmoth. The authors performed high-fidelity CFD modeling to predict the flapping wing aerodynamic forces, torque, and power of a hovering hawkmoth subjected to hybrid high- and low-fidelity models. The authors highlighted that the technique of combining CFD with quasi-steady modeling provides more accurate results compared to the blade element method subjected to *Re* varied by wing beat amplitude or frequency. Another study on hawkmoths is by Nguyen et al. [38], who study the aerodynamics of flapping wings using CFD simulations. The authors used the unsteady vortex-lattice method subjected to hovering flight, forward flight, and aerodynamic forces. From the results obtained, the aerodynamic forces and pitching moment showed good agreement with results from previous studies. It can also be noted that the forward flight at 2.1 ms^−^^1^ generated vertical force during the downstroke motion of the wings. Li et al. [39] were motivated by paired wings configuration and stroke-plane inclination in biological flapping flights of for optimal aerodynamic performance in quadrotor drone CFD-based analysis. By using ANSYS CFX, the authors simulated the aerodynamics of the rotor configuration subjected to tip distance, height difference, and tilt angle of the propellers and hence obtained an increased rate of lift force with 10.4% efficiency compared to the basic rotor configuration. The bio-inspired rotor configuration also provides improvements in terms of the height difference effect (4.3% in life and 8.4% in efficiency) compared to the basic rotor design. Yet, the tilt angle effect showed a minor effect in hovering conditions due to the stroke plane of the propellers’ reduced vertical force. Further, Li et al. [40] added a ducted multi-propeller design to improve the lift force and aerodynamic efficiency. Amazingly, the presence of ducting on the single propeller design improved lift force by 24.5% and efficiency by 38.1% compared to the non-ducted model. In fact, the aerodynamic interaction among ducted multi-propellers also had a significant dependency on tip distance and the height difference between propellers.

Besides that, the other type of insect of interest is the fruit fly. Zhang et al. [41] were inspired by hovering fruit flies in developing a flapping MAV wing model in hovering flight using CFD and ANSYS Fluent. The authors validated the study with previous experimental work and obtained good agreement in time-averaged lift coefficients. The author furthered the study by combining both methods of CFD and Taguchi and highlighting that the time-averaged lift forces are influenced by the flapping frequency and stroke amplitude. Jadhav et al. [42] compared CFD simulation with experimental studies of the flapping motion of flower flies. The authors studied the flow physics and flapping mechanism of MAV and concluded that the force measurement indicates the shorter distance between the wings at the end of the flapping motion, which provides a higher lift improvement for subsequent flapping motion. Thus, from the study, the authors suggested designing the MAV with the shortest possible distance between the leading edge of the flapping wing for the optimum advantage of the flapping mechanism. Nguyen et al. [43] conducted a CFD simulation to understand the aerodynamic performance of a fly-type flapping wing considering the control moments subjected to trailing edge change and stroke plane change. The stroke plane change provides better convergence speed, with an increase of 35% compared to the trailing edge change. At lateral motion, the trailing edge change showed a better improvement in convergence speed (44%), yet made the MAV slower compared to the stroke plane change. Thus, the stroke plane change demonstrated quicker response times with external disturbance and was selected for further MAV development. Olejnik et al. [44] used the immersed boundary method-based CFD simulation to investigate the aerodynamic performance of flying fruit flies by determining the temporal dynamics of the aerodynamic forces and torques during wingbeat. From the CFD data, the bio-inspired flapping fly-robot produced stable yaw torques due to the baseline positive stroke dihedral, especially when hovering or in slow forward flights. Perl et al. [45] were also inspired by fruit flying by evaluating the wingbeat-averaged aerodynamic forces and torques. The study is conducted by comparing both wings and one wing at a time to understand the wing-wing interaction that contributed to the negative sign of the sideways-roll coupling. Both wings showed that the aerodynamic forces and torques at the right wing of the fly during hover resulted in accurate wing kinematics and wing-wing interaction in the stability maneuver within 17 wingbeats.

### 3.3. Flying Animals

Ghosh [46] studied the flapping wing mechanism by estimating the amount of lift and drag produced by a flying bird known as the Thrush Nightingale in a steady flight using ANSYS Fluent software. In the CFD simulation, the author considered the body of the bird to be stationary while the wing was placed far away from the body due to the interference effect of the body. Furthermore, the wing kinematics calculation is also included to calculate the motion of the wings for the flapping mechanism. From the calculated 5 flaps of the wing, 0.4731 N was estimated in each flap, which can support the weight of the bird. The drag polarity of the flapping wing is subjected to the flow separation that developed in the low-pressure regions.

Another type of bird wing inspired by nature, known as the hummingbird, has also made progress in flapping wing UAVs due to its flapping mechanism and excellent characteristics of high agility and maneuverability, backward motion, long duration hovering, and being able to rotate their wings 180°. Kumar et al. [47] used ANSYS Fluent to perform fluid-structure interaction, where CFD simulation is conducted to study the aerodynamic performance of the wing of a giant hummingbird and compared it with the experimental study. It can be found that the wing was excited at 14 Hz, with negative drag indicated as positive thrust with high *Cl*. Geder et al. [48] used FEFLO software to perform CFD simulation on a flapping wing geometry inspired by a bird. The authors studied wing kinematics, aerodynamic forces, and pitch altitude control on the model of a flapping wing, in which both kinematics and flow environment affected the wing forces and captured the flow physics by using a reduced-order model. 40% of stroke average thrust decreased from forces of 0.5 N to 0.3 N. Yang et al. [49] also present a study on the numerical simulation of a bio-inspired flexible flapping wing of a dove’s flight abilities. By using ANSYS Fluent, the authors compare the aerodynamic forces of the flapping wing model between rigid and flexible wings subjected to vertical and horizontal directions. The authors further analyzed the flow study around the wing subjected to the downstroke and upstroke of the beginning and middle conditions. The flexible wing is great for thrust generation and obtaining more flow from the unsteady aerodynamics of the flapping wing in terms of vortex formation and evolution.

Bie et al. [13] investigated the aerodynamic characteristics at the design cruise of a bat-inspired tailless flapping wing UAV through CFD simulation. The author proved that the UAV is able to process an outdoor cruise, demonstrating that the size configuration is suitable for the design of a flapping-wing UAV. Comparing the numerical simulation with the test flight subjected to the same cruise speed resulted in the obtained flapping frequency and AoA having a difference of only 5.4% and 2.5%, respectively. Based on the results of CFD, it can be concluded that the bat-inspired UAV has the potential for higher take-off weights subjected to different conditions of frequency and AoA.

**Figure 5:**
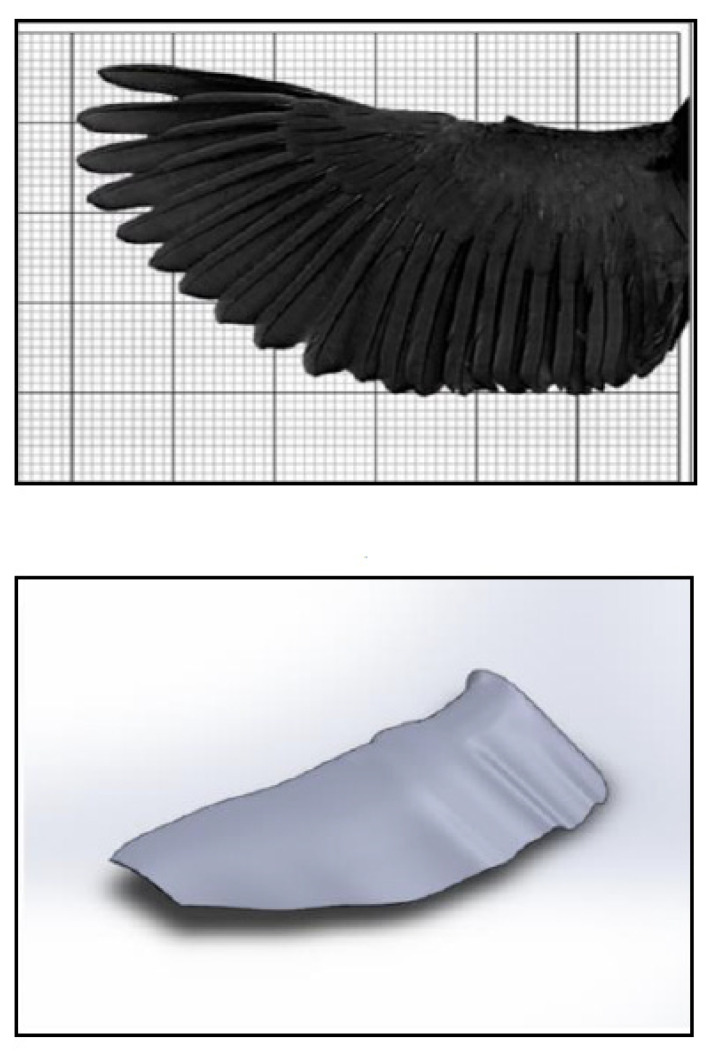
Mimicking the geometry of Kingfisher (reproduced with permission from [50]).

Abas et al. [50] demonstrated both CFD and experimental studies to investigate the aerodynamic performance of a Kingfisher-inspired flapping wing model (as in Figure 5) with a *Re* of 6024 at 4.4 m/s subjected to flapping frequencies of 11 Hz, 16 Hz, and 21 Hz. From CFD simulation using ANSYS Fluent, the lift produced by all AoA (0° to 20° at a 4° increment) proved that the same lift production cycle occurs during power flight and that negative lift is created at stroke reversal moments due to delayed rotation of the specified pitch kinematics, with a *Cl* of 3.45 at 12°. The drag forces showed increments in rotational angle increases, especially at the mid-forward and mid-backward strokes, subjected to all cases of flapping frequencies and angle instances. The study concluded that the Kingfisher bird produced significantly more lift on the downstroke than on the upstroke because of the edge vortices developed during the downstroke.

**Figure 6:**
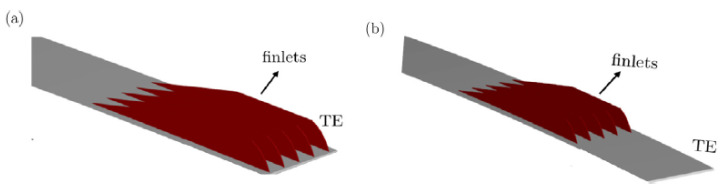
Finlet configuration (**a**) Bodling & Sharma [51] (**b**) Ananthan & Akkermans [52] (reproduced with permission from [52]).

Bodling and Sharma [51] performed a numerical analysis of an airfoil geometry inspired by the down coat of the night owl. The bioinspired geometry is an array of finlet “fences,” which apply at the trailing edge of the baseline NACA0012 airfoil. The authors used large eddy simulations to compare both baseline airfoil and bioinspired airfoil geometries (finlet fences) using FDL3DI software and further studied the aeroacoustic performance of the compared geometries using PEGASUS software. From the study, a large reduction at high frequency was found at the unsteady surface pressure near the airfoil trailing edge, but an increase at low frequency was found with the bioinspired airfoil. The presence of fences lifted the turbulence eddies away from the trailing edge, which reduced the scattering efficiency; hence, these findings are one of the mechanisms of noise reduction due to the increased source-scattering edge separation distance. Similar findings were found in the study by Ananthan and Akkermans [52], which were aimed to reduce the trailing edge noise using 2D finlets inspired by the owl, with the finlets placed upstream of the trailing edge. By using RANS/LES CFD simulation, the authors also used the baseline NACA0012 airfoil and compared the overset-large eddy simulations with the finlet’s configuration. From the study, the finlets mounted on the trailing edge of the airfoil revealed their dissipating effect on the surface pressure fluctuations, thus weakening the edge scattering. It can be observed that the flow velocity also decreased for the flow that exited the finlet channel towards the trailing edge. The difference between the two studies is illustrated in Figure 6.

Bardera et al. [53] performed a CFD analysis of a biomimetic UAV that mimicked the primary feathers of an eagle, known as wing grids. By using ANSYS Fluent, the study is conducted by comparing the aerodynamic efficiency for cruise and ascent phases subjected to the grid configurations (based on extension type and variable span type) at the wingtip. From the result, the extension of the grid wingtip only on one side proved to achieve a rolling moment of 0.06, especially for higher AoA, which is enough to maneuver. The flow visualization was also observed comparing both configurations, which improved the aerodynamic efficiency.

Gilberto et al. [54] performed CFD analysis to investigate the effect of the thermal difference between upper and lower surfaces subjected to the aerodynamic performance of the NACA 0012 airfoil, which is inspired by bird wings, using ANSYS Fluent. From the analysis, the heating of the lower surface at 50 °C differences showed an improvement in the lift while the drag coefficient remained nearly the same. Thus, the *L/D* ratio is improved in the majority of AoAs analyzed. The results help to consider heating devices on the wing of the UAV that could feasibly operate at low resolution. 

### 3.4. Aquatic Animals

On the other hand, aquatic animals are also no exception to biomimetics studies. In most studies, the part of the fins of aquatic animals commonly became the main interest in understanding the aerodynamic effects using CFD simulation. Replicating the natural body features and approach into CFD applications has the potential to contribute to aircraft wings or autonomous underwater vehicles.

**Figure 7:**
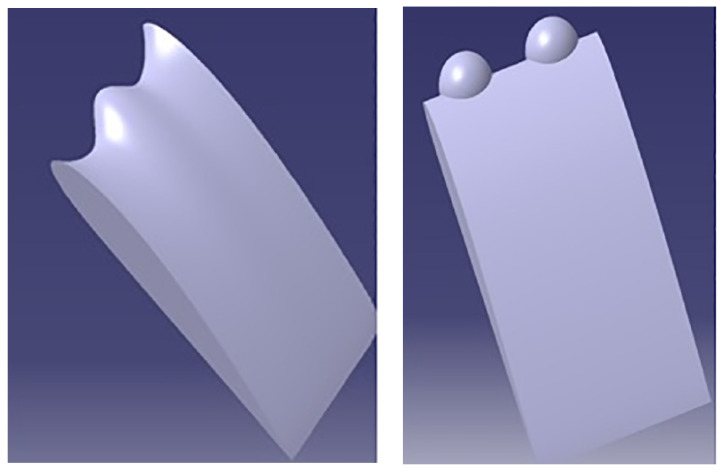
Tubercles configuration of sinusoidal and spherical mimicking the tubercles of a Humpback whale [19] (adapted with permission from PLoS ONE).

Aftab et al. [19] studied the aerodynamic effects of Humpback whale tubercles at the leading edge, which contribute to the reduction of separation bubble size and help in delaying stall, as in Figure 7. By using ANSYS Fluent, the authors simulated the tubercles at the leading edge of the sinusoidal and spherical patterns and compared the results of *Cl*, *Cd*, and *L/D* with the clean airfoil of NACA 4415. From the results, the spherical patterns performed the best aerodynamic effects at 18° AoA, with lift increasing by 5.96%, drag decreasing by 2.67%, and *L/D* improving by 6.25%. Arrondeau and Rana [55] investigated the impact of tubercles on the leading edge of a multi-element wing in ground effect by using the CFD tools of ANSYS Fluent. From the study, a significant improvement in aerodynamic performance was found at the front wing. The tubercle pattern more upstream impacted the flow and aerodynamic coefficients of the wing. Hence, an improvement of about 22.6% and 9.4% of the lift and *L/D* ratio for optimal amplitude and a number of tubercles with 167.7% stall delay, which proved that the presence of tubercles improved downforce generation while increasing drag. Papadopoulos et al. [56] performed a 3D numerical study on the influence of the spanwise distribution of tubercles on a UAV wing. By using ANSYS Fluent software, the lift increased with the addition of tubercle patterns subjected to AoA. The results showed a significant potential for controlling the flow on the wings of a UAV subjected to Re numbers ranging between 500,000 and 1,000,000, which improved the aerodynamic performance and efficiency.

Bhatia et al. [57] performed a CFD simulation on the surface of a 2D NACA 0012 airfoil. The authors compared the analysis with the experimental structure of the sharksin denticles to understand the aerodynamic performance, particularly in terms of rate of lift enhancement, rate of drag reduction, and *L/D* ratio subjected to variation in AoA. By using ANSYS Fluent software, the results showed that the maximum drag reduction and *L/D* enhancement were found to be improved by 3% and 1.5% at 0° AoA and 12° AoA, respectively. The presence of sharkskin denticles contributed to the damping of disturbance within the boundary layer, thus affecting the transition delay and drag reduction. Marimuthu et al. [58] also investigated the aerodynamic performance of a better surface design of a bio-inspired sharkskin pattern subjected to the aerofoil design of NACA 0012. The CFD simulation was conducted using ANSYS Fluent software and compared with the experimental results in terms of lift force and viscous drag. It can be observed that the result of using the sharkskin pattern on the NACA 0012 airfoil showed that lift force improved by 5.41% at 21° AoA and drag reduced by 9.98% at 15° AoA.

Meng et al. [59] developed a miniature macrofiber composite (MFC) robot fish inspired by koi fish. The authors performed both experiments and CFD simulations to study the thrust measurement system and propulsion performance subjected to thrust variation induced by the oscillating caudal fin of the robot fish. The propulsion performance of the robot fish showed a good agreement between both methods, thus proving that the thrust variations of the oscillating flexible structure actuated by MFC actuators are useful for the design and implementation of underwater biomimetic robots using smart actuators. Jaya and Rukmono [60] conducted a CFD analysis on a caudal fish fin propulsion for a small and low-speed autonomous underwater vehicle (AUV) by using ANSYS CFX. The authors studied the effects of variable thickness position and fin-base thickness on the normalized efficiency of a tapered fin. From the simulation, the flexible tapered fin showed an improvement of 25% compared to the narrow fin’s 30%. The visualization force vectors showed a greater thrust force in the thrust direction for flexible tapered fins. Thus, this result helps to develop an efficient AUV for the state’s defense, especially in the underwater region.

In summary, the overall literature on bio-inspired animals’ anatomical structures is reviewed based on the applications of CFD that relate to solving fluid behavior, in which the review is limited to at least 10 years of research (2013–2023). From the review, it can be concluded that the works are more focused on the use of CFD, including simulations and mathematical methods, considering other criteria such as dynamic variation mechanisms as the anatomical structures of animals influence aerodynamic characteristics. It can also be seen that understanding the fluid dynamics of a 3D geometry model is more complicated compared to a 2D model, considering the shape and aspect ratio and the completely distinct fluid dynamics. Hence, the effects of key parameters related to CFD analysis are crucial in defining the characteristics of fluid dynamic performance involving flying (using wings) and aquatic (using fins) animals. Thus, CFD plays an important role in simulating and imitating in real life the biological inspiration of nature for advanced technology development in the aerospace field.

## 4. CFD Model Construction

**Figure 8:**
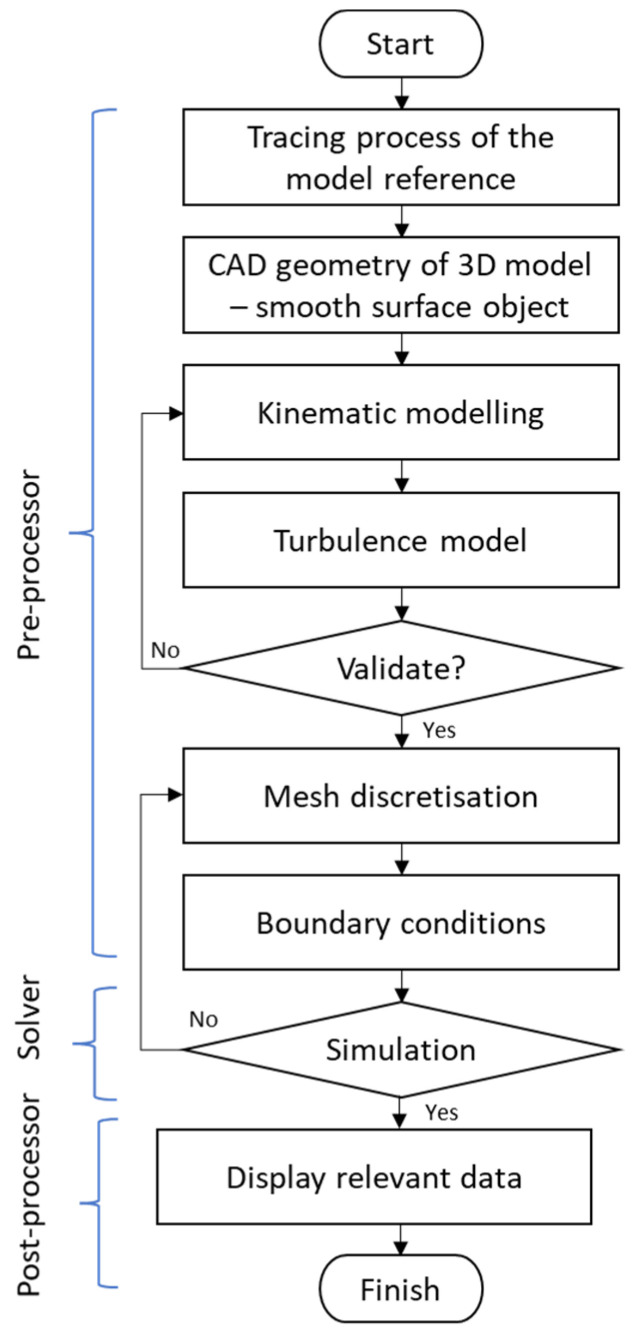
Sequential diagram of constructing CFD modeling.

Spalart and Venkatakrishnan [61] highlighted that CFD is increasingly being used in the multidisciplinary design and analysis of aerospace technologies. CFD is one of the most powerful tools for examining the behavior of a system, which is beneficial and leads to more innovation in the design of a system through the numerical overview of fluid flow. In the CFD methodology, it is commonly explained in terms of three main categories, known as pre-processor, solver, and post-processor. The procedure for constructing CFD modeling that can be applied to understanding the aerodynamic performance of bio-inspired applications is described in Figure 8.

Referring to Figure 8, the pre-processor is the modeling element as the input, which includes problem formulation, meshing, boundary condition setting, and generation of the computational model. The solver involves the processing elements, whereas the numerical solution methods involve discretized governing equations and algebraic solutions. The post-processor is the output element of the computational results that can be displayed and is subjected to the acceptable convergence of the equations that are being solved. In relation to the biomimetics study of CFD simulation, these three categories play a major role in ensuring the geometry definition, flow conditions, as well as appropriate discretization and boundary conditions, so that the flow field can be computed appropriately with sufficient accuracy in the region of the model. The most important step is mimicking or replicating the exact measurements of the anatomy of the bio-inspired animal, considering the features of anatomical mechanisms. The selection of a turbulence model is also crucial in order to justify the numerical investigation and the theory behind all the governing equations in the simulation. Then, the model is discretized into a finite set of control volumes to initiate numerical calculations and render solution fields using CFD simulation software.

### 4.1. Issues Related to CFD Study of Bio-Inspired Aquatic and Flying Animals

Flying animals, including birds and insects, take advantage of their flapping wings to hover and locomote in the air, which is produced by lift and thrust, while aquatic fish need to flap their pectoral and caudal fins to achieve optimal propulsion. This motion is known as the oscillatory motion of wings or fins. Behind the CFD simulation, the main issues are highlighted involving the theoretical knowledge of the governing body.

Governing Purview

Before performing CFD, it is important to understand the governing authority involved. As such, it can be categorized into four aspects: presumptive, geometrical, kinematic, and performance parameters.

#### 4.1.1. Presumptive Parameters

The presumptive parameters can be referred to as “environmental”, describing the properties of fluid that are supposed to be considered in flapping wings or fins. Parameters of the Reynolds number, Re, can be defined by dominant parameters in flapping airfoils such as freestream velocity, $U_{\infty }$, and kinematic viscosity of air/water, ν, as in Equation (1).

(1) $$Re=\frac{U_{\infty }L_{0}}{\nu }$$

where, $L_{0}$ is the characteristic length that is represented by the chord length, c, maximum thickness, *D,* or span length, *L*, especially for a 3D model of flapping foil. The corresponding *Re*, is as follows:

(2) $$Re_{c}=\frac{U_{\infty }c}{\nu },\;Re_{D}=\frac{U_{\infty }D}{\nu },\;Re_{L}=\frac{U_{\infty }L}{\nu }$$

The Mach number is also one of the key parameters in high-speed airflow, yet its effects on flapping wings are rarely mentioned.

Besides that, in solving CFD numerical simulations of flow behavior, the governing equations of Navier-Stokes and other conservative and non-conservative physics laws in mathematical language should be taken into account.

For continuity,

(3) $$\frac{\partial \rho }{\partial t}+\nabla .\left(\rho uV\right)=0$$

where, **V** is the velocity vector, and ρ is the density.

For momentum at *x, y,* and *z* components,

(4) $$x-component:\;\frac{\partial \left(\rho u\right)}{\partial t}+\nabla .\left(\rho uV\right)=-\frac{\partial p}{\partial x}+\frac{\partial \tau_{xx}}{\partial x}+\frac{\partial \tau_{yx}}{\partial y}+\frac{\partial \tau_{zx}}{\partial z}+\rho f_{x},\;y-component:\;\frac{\partial \left(\rho v\right)}{\partial t}+\nabla .\left(\rho uV\right)=-\frac{\partial p}{\partial y}+\frac{\partial \tau_{xy}}{\partial x}+\frac{\partial \tau_{yy}}{\partial y}+\frac{\partial \tau_{zy}}{\partial z}+\rho f_{y},\;z-component:\;\frac{\partial \left(\rho w\right)}{\partial t}+\nabla .\left(\rho uV\right)=-\frac{\partial p}{\partial z}+\frac{\partial \tau_{xz}}{\partial x}+\frac{\partial \tau_{yz}}{\partial y}+\frac{\partial \tau_{zz}}{\partial z}+\rho f_{z},$$

where *p* is pressure, $\tau_{ii}$ denotes stress. In addition, the velocity vector is decomposed in *x*, *y,* and *z* components denoted as *u*, *v,* and *w*, respectively.

While, for energy conservation equations, as follows:

(5) $$\frac{\partial }{\partial t}\left[\rho \left(e+\frac{V^{2}}{2}\right)\right]+\nabla .\left[\rho \left(e+\frac{V^{2}}{2}V\right)\right]\;\;\;\;\;\;\;\;\;\;\;\;\;\;\;\;\;\;\;\;\;\;\;\;\;=\rho \dot{q+}\frac{\partial }{\partial x}\left(k\frac{\partial T}{\partial x}\right)+\frac{\partial }{\partial y}\left(k\frac{\partial T}{\partial y}\right)+\frac{\partial }{\partial z}\left(k\frac{\partial T}{\partial z}\right)-\frac{\partial \left(up\right)}{\partial x}-\frac{\partial \left(vp\right)}{\partial y}\;\;\;\;\;\;\;\;\;\;\;\;\;\;\;\;\;\;\;\;\;\;\;\;\;--\frac{\partial \left(wp\right)}{\partial z}+\frac{\partial (\partial u\tau_{xx)}}{\partial x}+\frac{\partial \left(\partial u\tau_{yx}\right)}{\partial y}+\frac{\partial \left(\partial u\tau_{zx}\right)}{\partial z}+\frac{\partial \left(\partial v\tau_{xy}\right)}{\partial x}\;\;\;\;\;\;\;\;\;\;\;\;\;\;\;\;\;\;\;\;\;\;\;\;\;\;+\frac{\partial \left(\partial v\tau_{yy}\right)}{\partial y}+\frac{\partial \left(\partial v\tau_{zy}\right)}{\partial z}+\frac{\partial \left(\partial w\tau_{xz}\right)}{\partial x}+\frac{\partial \left(\partial w\tau_{yz}\right)}{\partial y}+\frac{\partial \left(\partial w\tau_{zz}\right)}{\partial z}\;\;\;\;\;+\rho f.V$$

where $\dot{q}$ is denoted as the volumetric heat addition, e is the specific energy f represents field forces such as gravity, k is the thermal conductivity, and T is temperature.

#### 4.1.2. Geometrical Configuration

In some research work, the geometry mimicking the features of animals can be created in 2D or 3D. For the 2D model, most studies were conducted due to the advantages of a simple problem, easy mathematical description, clear physics, or a simple numerical solution [62].

In mimicking flying and aquatic animals, it is important to create a realistic 3D model of a wing or fin, incorporating the shape, angular dispersions, and wing morphological characteristics such as area, mean chord, length, etc., and modeling it into CAD geometry. Moreover, the reconstruction of the wing/fin surface is also important during the creation of the model as it affects any further numerical simulation setup.

In some other works, the effect of aspect ratio (*AR*) on 3D flapping wings/fins is considered. The *AR* is defined as:

(6) $$AR=\frac{L}{c}$$

where *L* is the span length and *c* is the chord length.

Besides that, the flexibility of the wing/fin foils should also be considered, as the shape of the wing/fin may change with its flapping motion. As in the literature, most of the studies presented in the geometry, including the airfoil and AoA at which the airfoil is submitted considering the free stream velocity and boundary conditions.

#### 4.1.3. Kinematic Modeling Parameters

The morphological and kinematic models of both flying and aquatic animals have the same dynamic variation mechanisms when subjected to fluid mediums such as air or water. In most studies reviewed, the simulation of CFD was mostly based on dynamic kinematic analysis subjected to oscillation motion. According to Singh et al. [11], the unsteady flow dynamics induced the oscillation of aerodynamic force and moments of flying animals; thus, a mathematical model with nonlinearity should be considered. Abas et al. [50] highlighted the use of predictive quasi-steady model approximation for the condition of unsteady aerodynamic forces that influenced rotation, translation, rotation-translation coupling, and the added-mass effect. The motion of the wing or fin can be implemented using time histories of attitude angles rotated with respect to three different axes of the local coordinate systems.

The governing equation describing the oscillation motion can be characterized by the Sthrouhal number, the *St* of the externally imposed frequency, and *Re*. For the non-dimensional format of incompressible flow using the Navier-Stokes:

(7) $$St\frac{\partial u}{\partial Ƭ}+u.\nabla u=-\nabla p+\frac{1}{Re}\nabla^{2}u$$

(8) ∇.u=0

where, u, p and Ƭ are the non-dimensional flow velocity, pressure, and time, respectively.

(9) $$St=\frac{c}{\emptyset r_{2}}\;or\;\frac{fA}{U_{ref}}$$

where *c* is the mean chord length, $r_{2}$ is the radius gyration of the wing, A is the peak-to-peak oscillation amplitude, and stroke period *T* = 1/*f*. Therefore, $U_{ref}=2\emptyset r_{2}$.

Other important factors should be considered, such as reference velocity, stroke frequency, and position at a given time. For 3D angular flapping motions, the relationship between flapping angles and variation with stroke cycle, as well as rotation, are the parameters to be taken into account for any kind of numeric analysis.

#### 4.1.4. Performance Parameters

The parameters describing the performance of fluid dynamic characteristics include lift and drag coefficients for aerodynamic wings, and thrust coefficient, input power, and efficiency for propellers.

The force coefficients are the main parameter used to assess the influence of the different wing motion models.

The lift coefficient, *Cl*, is defined as:

(10) $$Cl=\frac{F_{lift}}{\bar{q\;}\cdot S}$$

The drag coefficient, *Cd*, is:

(11) $$Cl=\frac{F_{drag}}{\bar{q\;}\cdot S}$$

where, *S* is the wing surface and $\bar{q\;}$ is the mean dynamic pressure, which can be obtained by:

(12) the q¯=12ρV2ref=12ρ·1T∫0TV∞+Vflapt2+Vdevt2dt

where the integration is calculated based on one flapping cycle with period *T* [s]. The reference velocity contains the free-stream velocity $V_{\infty }$, which is non-zero in forward flapping flight; the flapping velocity ($V_{flap}$ and deviation velocity ($V_{dev}$ perpendicular to the horizontal plane (in hovering flight).

Another interesting parameter to indicate aerodynamic performance is in terms of thrust coefficient $\bar{C_{T}}$, input power coefficient $\bar{C_{P},}$ and efficiency, *η*, are mainly for propulsion.

(13) CT¯=F¯12ρV2∞cL

where, $\bar{F}$ is a time-averaged drag force, which is defined by:

(14) $$\bar{F}=\frac{1}{T}\int_{0}^{T}F\left(t\right)dt$$

where, $F\left(t\right)$ is the instantaneous force component in the *x* or *y* direction, and *T* is the oscillation period.

The mean input power coefficient $\bar{C_{P}}$ is:

(15) CP¯=P¯12ρV3∞cL

where the input power, $\bar{P}$, can be calculated by:

(16) $$\bar{P}=\frac{1}{T}\left[\int_{0}^{T}F_{lift}\left(t\right)\frac{dh\left(t\right)}{dt}dt+\int_{0}^{T}M_{\theta }\left(t\right)\frac{d\theta \left(t\right)}{dt}dt\right]$$

where, $F_{lift}\left(t\right)$ is the instantaneous force component of lift, $M_{\theta }\left(t\right)$ is the instantaneous pitching moment, $\frac{dh\left(t\right)}{dt}$ and $\frac{d\theta \left(t\right)}{dt}$ are derivatives of lift and pitch motion, respectively.

Hence, the efficiency of propulsion can be expressed as:

(17) $$\eta =\frac{\bar{C_{T}}}{\bar{C_{P}}}$$

Therefore, it must be noted that the key parameters describing the performance of aerodynamics for flapping wings, fins, or propulsion applications are under the conditions of an oscillating wing or fin subjected to force and moment. The mathematical expression can be expressed for 2D models by incorporating the direction of x and y components. Thus, the aerodynamic performance in CFD simulation is mostly analyzed based on kinematic models with different degrees of complexity based on mimicked flying or aquatic animals.

### 4.2. Merits and Limitations of Biomimetics-CFD Applications

CFD has received attention from mathematical curiosity and has become an important technique to study complex physical flow patterns and demonstrate their potential, especially in bio-inspired systems. To date, CFD has been adopted by most researchers to investigate the characteristics of fluid flow subjected to the anatomical structure of bio-inspired applications. Hence, it offers benefits such as quick assessment of design variations, facilitation in understanding comprehensive information to interpret the performance, conveying a good understanding of physical mechanisms, and simulation of different conditions. From a theoretical point of view, CFD provides benefits by focusing on modeling and solving the governing equations, as well as investigating the number of approximations used for these equations. Meanwhile, both numerical and experimental techniques highlighted the merits of CFD as an alternative to cost, which is that CFD simulations are relatively inexpensive and will become less expensive as computers become more powerful.

Despite that, CFD still faces several limitations that need to be addressed. In the design and creation of databases as well as in multi-disciplinary applications, the turnaround time associated with CFD has become one of the main factors that limit its use of CFD. This involves the geometry, which may contain gaps, multiple definitions, and intersecting surfaces required to be resolved. The problem of clean geometry specification is more pressing and delicate when meshing techniques are employed. Another limiting factor is the level of skills required of the user of CFD, as CFD involves geometry preparation, meshing, solution setup, and post-processing that require a long lead time. Besides that, the accuracy of the numerical solution is one of the main challenges in applying CFD that is related to numerics, physical modeling (especially transition and turbulence), and the time involved in preparing geometries for conducting mesh generation and aerodynamic analyses. The challenge for CFD is how to adapt to newly emerging architectures such as processors, which may slow the growth of computing power. Besides that, the accuracy of physical modeling became the biggest challenge in performing CFD. This is because the turbulence is quite complex as it requires several steps and is fully integrated with the grid design; this is unaesthetically pleasing.

## 5. Conclusions

The rapid development of outstanding aerospace and similar industries has led to the substitution of CFD in the analysis, design, certification, and support of aerospace products. Due to the recent advancement in computational technology, numerical solutions for physically and geometrically complex systems, especially those mimicking the features of bio-inspired animals, can also be evaluated using CFD techniques. Besides becoming faster and more affordable by exploiting higher computing power, CFD has become more reliable, more reproducible across users, and better understood and integrated with other disciplines and engineering processes. Therefore, it is important to demonstrate the effectiveness of simulation results relative to the actual mechanisms of animal anatomical features through numerical solutions and physical modeling. The widespread acceptance of aerospace design and analysis, primarily by CFD, will be a remarkable achievement and bring benefits to users, especially researchers and engineering practitioners.

## Figures and Tables

**Table 1:** Biomimetics studies in engineering applications.

Category	Animal	Anatomical Structures	Anatomical Advantages	Engineering Application	References
Flying	Birds	Limbs and feathers	The forces of lift, thrust, drag, and gravity influence the flight patterns of birds	Flapping UAVs	[7]
	Owls	Feathers	Fly silently and help to absorb aerodynamic sound and suppress vibrations when waves of sound come crushing over the wing	UAVs Wind turbines	[7]
	Wild geese	Wings	Ascending air current with less effort	AIRBUS	[8]
	Insects (dragonfly)	Multiple wings and legs	Pressure gradients for lift and thrust by flapping	small UAVs (micro aerial vehicles)	[9]
		Flapping wings	Wake capture occurs when wings change direction	Biomimetic MAV	[10]
	Insects (mosquito)	Flapping wing and membrane wing	Flaps at a moderately high frequency relative to similar insects	Miniature unmanned autonomous (robots)	[11]
	Bats	Limbs	The membrane of skin that stretches between arms and legs helps to produce lift	small UAVs (micro aerial vehicles)	[9,12,13]
Aquatic	Whale	Flipper (Tubercles effect)	Tubercles on the leading edge produce greater lift and less drag than a smooth surface fin	small UAVs (micro aerial vehicles)	[14,15,16,17,18,19]
	Tuna	Median fins	Hydrofoils produce sideways lift force when the fin plane is at an angle with the fluid flow direction	Autonomous underwater vehicles	[20,21]
